# Cyclin B2 (CCNB2) Stimulates the Proliferation of Triple-Negative Breast Cancer (TNBC) Cells *In Vitro* and *In Vivo*

**DOI:** 10.1155/2021/5511041

**Published:** 2021-07-26

**Authors:** Shuai Wu, Rui Su, Hongyan Jia

**Affiliations:** ^1^Department of Mammary Gland, The First Hospital of Shanxi Medical University, Taiyuan 030001, China; ^2^Department of Critical Care Medicine, The Second Hospital of Shanxi Medical University, Taiyuan 030001, China

## Abstract

Triple-negative breast cancer (TNBC) is the most aggressive type of breast cancer. Currently, targeting therapy makes great advances for the treatment of TNBC, whereas more effective therapeutic targets are urgently needed. Cyclin B2 (CCNB2), which belongs to B-type cyclins, is known as a cell cycle regulator. CCNB2 is synthesized at G1 phase in cancer cells and downregulated at anaphase. The defects of CCNB2 led to the abnormal cell cycle and tumorigenesis. Though there are wide effects of CCNB2 on multiple types of tumors, the potential role of CCNB2 in TNBC progression is still unclear. Herein, we found that CCNB2 was highly expressed in human TNBC tissues and correlated with the prognosis and clinical pathological features including tumor size (*p* = 0.022^∗^) and pTNM stage (*p* = 0.021^∗^) of patients with TNBC. CCNB2 could promote the proliferation of TNBC cells *in vitro* and in mice. Our findings therefore confirmed the involvement of CCNB2 in TNBC progression and provided the evidence that CCNB2 could serve as a promising molecular target of TNBC.

## 1. Introduction

Triple-negative breast cancer (TNBC), which accounts for approximately 15% total incidence of breast cancer, is more aggressive than other types of breast cancers [1]. TNBC was highly metastatic, suggesting that TNBC was prone to recurrence average 2 years after surgery [2]. Notably, the standard treatment methods for TNBC mainly included surgery, chemotherapy, radiation treatment, and combination treatment. However, most TNBC patients still died within 5 years after therapy [3]. Currently, targeted therapy has great advantages for the treatment of high-malignancy tumors, particularly TNBC [4]. To combat this disease, more effective molecular targets are urgently needed.

The cell cycle is tightly and precisely regulated by a variety of regulators, and its abnormalities might lead to tumorigenesis. During the cell cycle, cyclins could bind to the cyclin-dependent kinases (CDKs) to affect expression and the activity of CDKs, therefore mediating cell cycle [5]. Cyclin B2 (CCNB2), which belongs to B-type cyclins, is a cell cycle regulator [6]. A previous study indicated that CCNB2 was synthesized at G1 phase and quickly downregulated at anaphase [7]. During cell cycle, the defects of CCNB2 led to the failure of G2/M checkpoint and stimulated gene mutations and tumorigenesis [8]. CCNB2 was also involved in meiosis progression in mouse oocytes [9].

The role of CCNB2 in cancer progression and metastasis has also been widely revealed [10, 11]. CCNB2 was abnormally expressed in several types of cancers, such as lung cancer, bladder cancer, and breast cancer [11, 12]. Its overexpression led to the poor prognosis of patients with hepatocellular carcinoma (HCC) [13]. A microRNA, miR-582-3p, could suppress the proliferation of acute myeloid leukemia via targeting CCNB2 [14]. Though there are wide effects of CCNB2 on cancer progression, its potential role in breast cancer is still unclear. Currently, there is no effective therapeutic target for the treatment of TNBC, a type of breast cancer which is highly metastatic. Targeting cell cycle regulators may be an effective method to combat TNBC [13]. Various cell cycle regulators have previously been found to affect TNBC progression and could serve as potential targets [14]. However, the effect of cyclins on TNBC progression is still worth further study.

This study was aimed at assessing the expression of CCNB2 in human TNBC tissues and investigated the correlation between its expression and the prognosis and clinical features of TNBC patients. We further detected the effects of CCNB2 on the proliferation of TNBC cells *in vitro* and *in vivo* and assessed the effects of CCNB2 in on tumor growth of TNBC cells. We therefore provided a promising molecular target for TNBC treatment.

## 2. Materials and Methods

### 2.1. Antibodies, Primers, and shRNA Plasmids

Anti-CCNB2 antibody (for IHC assays, 1 : 500 dilution, for immunoblot assays, 1 : 1000 dilution, ab6185622, Abcam, Cambridge, UK) and anti-*β*-actin antibody (1 : 2000 dilution, ab8226, Abcam, Cambridge, UK) were used.

The quantitative PCR primer sequences of CCNB2 are shown as follows: forward, 5′-CAACCCACCAAAACAACA-3′ and reverse, 5′-AGAGCAAGGCATCAGAAA-3′; the quantitative PCR primer sequences of GAPDH are shown as follows: 5′-TGACTTCAACAGCGACACCCA-3′ and 5′-CACCCTGTTGCTGTAGCCAAA-3′. CCNB2 shRNA plasmids were bought from Addgene. For CCNB2 depletion, cells were transfected with control or CCNB2 shRNA plasmids using Lipofectamine 2000 (Invitrogen; Thermo Fisher Scientific, Inc.), and the *in vitro* assays were performed 24 h after the transfection.

For the infection of lentivirus, lentivirus packaging plasmids were all purchased from Shanghai HanBio Co. Ltd. The virus was packaged in a 6-well plate; 1.5 *μ*g packaged mixed plasmid and 0.5 *μ*g expressed plasmid were added, together with 250 *μ*L serum-free medium. HEK293T cell density reached 90% for virus encapsulation. Subsequently, ultracentrifugation was used to harvest viral supernatant, and all follow-up experiments were carried out according to the instruction manual.

### 2.2. Bioinformatics Analysis

We conducted bioinformatics analysis through GEPIA (http://gepia.cancer-pku.cn/detail.php?gene=CCNB2/) to analyze the Cancer Genome Atlas (TCGA) database (the Ensemble ID is ENSG00000157456.7) with a threshold of *p* < 0.05 and LogFC > 1 or <-1 for the differential genes, and the median was used as the basis for dividing the patients into two groups for Kaplan-Meier (KM) survival analysis, and the 95% confidence interval was marked with a dotted line.

### 2.3. Human Tissue Samples and Analysis

The total 114 human TNBC tissues and corresponding normal tissues in this study were collected from the patients receiving surgical treatment in the First Hospital of Shanxi Medical University from 2016.6 to 2019.1. The corresponding normal tissues were adjacent normal tissues 3 mm far from tumor tissues of TNBC patients. Our study was approved by the Ethics Committee of the First Hospital of Shanxi Medical University, and all patients were informed of the study content and signed a consent form. The clinical-pathological features, including patient age, tumor grade, tumor size, pTNM stage, and lymph node metastasis, are listed in Table 1.

To explore the possible correlations between the expression levels of CCNB2 and TNBC progression, immunohistochemical (IHC) assays were performed. Briefly, tissues were paraffin-embedded using Leica paraffin embedding agent. The slices were sliced in 5 microns thick. Then, sections were fixed with 4% PFA at room temperature for 30 min at room temperature and subsequently blocked with 2% BSA for 20 min at room temperature. Slides were incubated with CCNB2 antibodies (1 : 500 dilution, ab6185622, Abcam, Cambridge, UK) at room temperature for another 2 h. After washing with PBS, the sections were incubated with biotinylated secondary antibody for another 1 h at room temperature (1 : 500, cat. no. ab201485; cat. no. ab99807, respectively; Abcam), and diaminobenzidine was used as a chromogen substrate. Finally, Leica EVOS fluorescence microscopy is used for immunohistochemical imaging.

CCNB2 was located in the cytoplasm of TNBC tissues. The scoring methods were shown as follows: 0: 0% stained cells; 1: 1–20% stained cells; 2: 21–60% stained cells; and 3: 61–100% stained cells. The staining intensity was evaluated on a score of 0 (negative or low-level staining), 1 (modest-level staining), and 2 (high-level staining). The expression levels of CCNB2 were divided according to the staining index: score of staining intensity + score of staining cells percentage. Staining index < 2 was thought to be low expression, while staining index 2 or >2 was thought to be high expression.

### 2.4. Cell Culture and Transfection

The human TNBC cell lines, MDA-MB-231 and HCC-1937, were all bought from ATCC. MDA-MB-231 and HCC-1937 cells were all maintained in Dulbecco's modified essential medium (DMEM), supplemented with 10% fetal bovine serum (FBS, Gibco, CA, USA), in a 5% CO_2_ incubator. The CCNB2 shRNA plasmids were transfected into both MDA-MB-231 and HCC-1937 cells by Lipofectamine 2000 (Invitrogen, CA, USA). The CCNB2 stably depleted MDA-MB-231 cells were manually screened through the infection of its shRNA plasmids and further used for the *in vivo* tumor growth assays.

### 2.5. Quantitative PCR Assays

Trizol (Invitrogen; Thermo Fisher, USA) was used to isolate total RNA from two types of TNBC cell lines. RNA was reverse transcribed using M-MLV reverse transcriptase (M1701; Promega). Total mRNA was reverse transcribed to produce cDNA using a cDNA synthesis system (Takara, Japan). qPCR was performed using a SYBR Ex Taq kit (Takara, Japan), which was used based on the manufacturer's protocol. The reaction conditions were as follows: predenaturation, 95°C, 5 min; denaturation, 95°C, 30 s; annealing, 58°C, 30 s; and extension, 72°C, 30 s. There were a total of 35 cycles. The method of 2 − ΔΔCq Livak and Schmittgen was used. CCNB2 mRNA levels were normalized to GAPDH.

### 2.6. Immunoblot Assays

TNBC cells or tissues were lysed by RIPA. BSA method was used for the determination of total proteins, and 15 *μ*L protein sample was loaded in the lane at a protein concentration of 1 mg/mL. Then, SDS-PAGE (12% gel) was performed. After adding the transmembrane onto NC membranes, membranes were blocked with 5% fat-free milk in TBST at room temperature and subsequently incubated with the primary antibodies of CCNB2 and *β*-actin at room temperature for 1.5 h. Then, the NC membranes were incubated with HRP-conjugate secondary antibodies for 1 h at room temperature. Signals were detected using ECL kit (Novex™ Chemiluminescent Substrate Reagent Kit, Thermo Fisher).

### 2.7. Colony Formation Assays

TNBC cells were added into a 6-well culture plate and transfected with control or CCNB2 shRNA plasmids. After 2 weeks, cells were fixed with PFA for 30 min at room temperature and stained with 0.1% crystal violet at room temperature for 20 min, then washed with PBS twice. The number of colonies was manually counted.

### 2.8. MTT Assays

TNBC cells were plated into 96-well plates with a density of approximately 1000 cells, transfected with control or CCNB2 shRNA plasmids, and maintained for 24 h. Cells were then incubated with MTT for 4 h at room temperature and then removed the medium. 150 *μ*L dimethyl sulfoxide (DMSO) was added into each well to extract the cells, and the absorbance value was measured with a microplate reader at 570 nm wavelength.

### 2.9. Tumor Growth Assays

All animal assay processes were approved by our Institutional Animal Care and Use Committee (IACUC) of the First Hospital of Shanxi Medical University (Approval number: SYXK 2019-0721). Bulb/C nude mice were purchased from Viton Lihua, Beijing. The mice were all female mice (8 weeks, 20-22 g). The mice were fed with adequate food and water, alternating 12 h of light and darkness, and their status was checked twice a day. There were 8 mice in the control group and 8 mice in the experimental group. Mice with tumors up to 1000 mm^3^ were considered to be killed. The tumor tissues were sacrificed by neck breaking before removal.

To generate stable CCNB2-depletion TNBC cells, the pGFP-V-RS-shCCNB2 plasmid and the packaging plasmids pVSVG and pMLV-Gag-Pol were transfected into HEK293T cells for 48 h. The virus-containing supernatant was harvested and filtered and concentrated using ultracentrifugation and transduced into MDA-MB-231 cells with polybrene (Sigma-Aldrich). Stable CCNB2-knockdown cells were then screened by puromycin (Invitrogen).

Subsequently, about 10^6^ MDA-MB-231 cells stably transfected with the indicated shRNA plasmids were subcutaneously implanted into athymic nude mice. After 14 days, tumors began formation, and the volume of tumors was measured every 3 days. After 29 days, all tumors were isolated, and the tumor growth curves were calculated and analyzed.

### 2.10. Statistical Analysis

GraphPad 6.0 was used in this study for all statistical analysis in this study. The results in this study were represented as mean ± SD. The correlations between clinical pathological features of TNBC patients and CCNB2 expression were analyzed using *χ*^2^ analysis. Student's *t*-test was used for statistical comparisons. ∗ indicated that *p* < 0.05 and was considered as a statistically significant difference.

## 3. Results

### 3.1. CCNB2 Was Highly Expressed in Human TNBC Tissues and Correlated with the Prognosis of TNBC Patients

**Figure 1:**
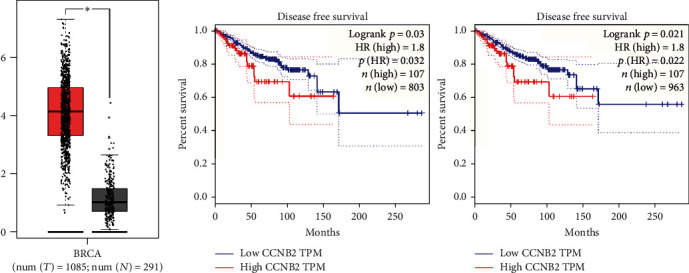
The CCNB2 mRNA level was upregulated in TNBC tissues and correlated with the prognosis of TNBC patients. (a) CCNB2 mRNA level in 1085 TNBC tissues was significantly higher than that in 291 normal tissues according to TCGA database. (b) CCNB2 expression level was associated with the disease-free survival rates of two groups of TNBC patients (*n* = 803 and *n* = 963, respectively). Results are presented as mean ± SD, ^∗∗^*p* < 0.01.

Since there are wide effects of CCNB2 on the progression of multiple types of cancers, we therefore speculate that CCNB2 has a potential regulatory role in the progression of TNBC. We investigated the expression levels of CCNB2 in human breast cancer tissues according to TCGA database. A total of 1085 tumor tissues and 291 normal tissues were used to assess the expression of CCNB2 in these tissues. We noticed CCNB2 expression was obviously enhanced in tumor tissues (Figure 1(a)). Furthermore, through the K-M survival analysis in TCGA database, we found that the expression of CCNB2 was obviously correlated with the disease-free survival rates of 2 sets of patients with breast cancer collected in different times (Figure 1(b), *n* = 803 and *n* = 963, respectively). Therefore, these data indicated the high expression of CCNB2 in human breast cancer tissues and correlated with the prognosis of patients.

### 3.2. CCNB2 Expression Was Upregulated in Human TNBC Tissues and Correlated with the Clinical Pathological Features of TNBC Patients

**Figure 2:**
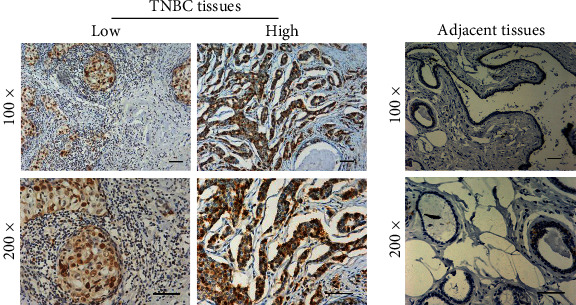
CCNB2 expression was enhanced in human TNBC tissues. (a) Immunohistochemical (IHC) assays were performed, and the CCNB2 expression levels in human TNBC tissues were shown (100x and 200x magnification, respectively). (b) IHC assays confirmed the relative low expression of CCNB2 in the corresponding adjacent tissues (100x and 200x magnification, respectively); scale bar indicates 100 *μ*m.

We then performed IHC assays to further evaluate the expression of CCNB2 in a total of 114 TNBC tissues and the corresponding adjacent tissues collected in our hospital. We found that CCNB2 was mainly located in the cytoplasm (Figure 2). Importantly, we also noticed that CCNB2 was highly expressed in TNBC tissues (Figure 2(a)), compared to normal tissues (Figure 2(b)). Our data therefore confirmed the high expression of CCNB2 in human TNBC tissues.

We subsequently analyzed the correlations between CCNB2 expression and clinical pathological features of patients with TNBC. The total of 114 patients was divided into CCNB2 low- and high-expression groups according to the expression levels of CCNB2 in tumor tissues; we noticed that 46 patients showed CCNB2 low expression (40.3%, Table 1), and the remaining 68 patients (59.7%) showed high CCNB2 expression. The clinical features, including patient age, tumor grade, tumor size, pTNM stage, and lymph node metastasis, were analyzed. We found no obvious correlations between CCNB2 expression and patient age (*p* = 0.141), tumor grade (*p* = 0.160), and lymph node metastasis (*p* = 0.247) of TNBC patients. Importantly, we noticed that CCNB2 expression was obviously correlated with tumor size (*p* = 0.022^∗^) and pTNM stage (*p* = 0.021^∗^) of patients with TNBC.

### 3.3. CCNB2 Promotes the Proliferation of TNBC Cells In Vitro

**Figure 3:**
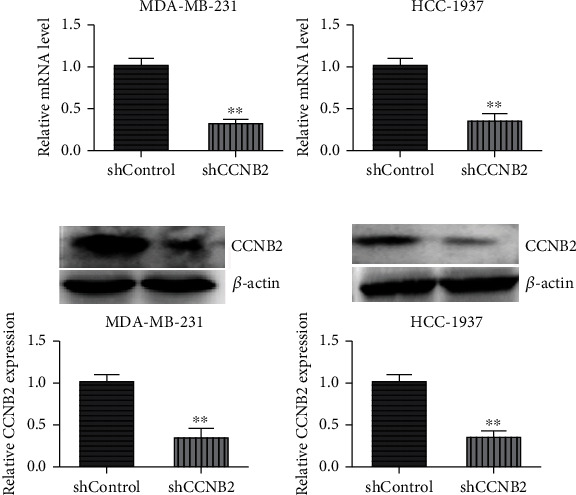
CCNB2 expression was decreased in both MDA-MB-231 and HCC-1937 cells after the transfection of its shRNA plasmids. (a) Quantitative PCR assays exhibited the decreased mRNA levels of CCNB2 after the transfection of its shRNA plasmids in MDA-MB-231 and HCC-1937 cells, respectively. (b) Immunoblot assays showed the decrease of CCNB2 expression after the transfection of CCNB2 shRNA plasmids in both MDA-MB-231 and HCC-1937 cells. Results are presented as mean ± SD, ^∗∗^*p* < 0.01.

Due to the high expression of CCNB2 in human TNBC tissues, we next performed the *in vitro* assays to investigate the possible role of CCNB2 in TNBC progression. The shRNA plasmids of CCNB2 were transfected into two types of TNBC cell lines, MDA-MB-231 and HCC-1937, to deplete its expression. We detected the expression of CCNB2 in MDA-MB-231 and HCC-1937 cells after the transfection of control or CCNB2 shRNA plasmids through quantitative PCR assays and immunoblot assays. The results of quantitative PCR assays confirmed that CCNB2 mRNA levels were significantly decreased after the transfection of its shRNA plasmids in MDA-MB-231 and HCC-1937 cells, respectively (Figure 3(a)). Similarly, a decrease of CCNB2 protein levels was also found in CCNB2 shRNA-transfected MDA-MB-231 and HCC-1937 cells, respectively (Figure 3(b)).

**Figure 4:**
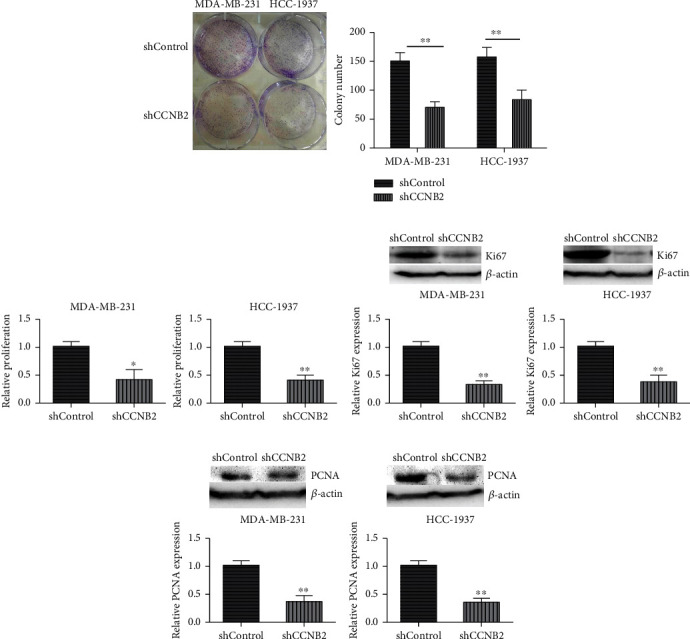
CCNB2 ablation suppressed the proliferation of TNBC cells *in vitro*. (a) Colony formation assays were performed by the use of MDA-MB-231 and HCC-1937 cells transfected with control or CCNB2 shRNA plasmids, and the number of colony in each group was counted. (b) MTT assay results showed the decrease OD value at 570 nm wavelength following CCNB2 depletion. (c) Ki67 expression levels in MDA-MB-231 cells and HCC-137 cells upon the indicated treatment. (d) PCNA expression levels in MDA-MB-231 cells and HCC-1937 cells upon the indicated treatment. Results are presented as mean ± SD, ^∗^*p* < 0.05 and ^∗∗^*p* < 0.01.

Subsequently, the colony formation and MTT assays were performed to assess the effects of CCNB2 on the proliferation of TNBC cells. After the depletion of CCNB2, an obvious decrease of colony number in both MDA-MB-231 and HCC-1937 cells was found through colony formation assays (Figure 4(a)). Similarly, through MTT assays, we also noticed that CCNB2 knockdown led to the decrease of cell proliferation in MDA-MB-231 and HCC-1937 cells, respectively (Figure 4(b)). In addition, we noticed that the expression of two cell proliferation markers, Ki67 and PCNA, is obviously decreased after CCNB2 depletion in MDA-MB-231 and HCC-1937 cells.

### 3.4. CCNB2 Contributed to Tumor Growth of TNBC Cells in Mice

**Figure 5:**
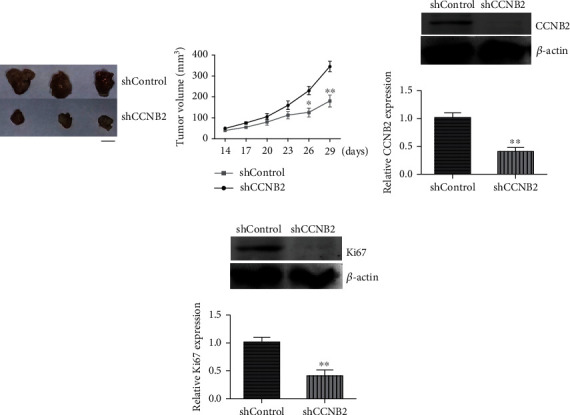
CCNB2 promotes tumor growth of TNBC cells in mice. (a) MDA-MB-231 cells infected with control or CCNB2 shRNA plasmids were subcutaneously implanted into nude mice. After 14 days, tumors began formation, and the volume of tumor in each group was measured every 3 days. After 29 days, all tumors were isolated, and the growth curves were shown. (b) Immunoblot assays showed the expression levels of CCNB2 in control or CCNB2 depletion tumors from mice. Results are presented as mean ± SD, ^∗^*p* < 0.05 and ^∗∗^*p* < 0.01. Scale bar indicates 5 mm.

To further confirm our previous *in vitro* results, we detected whether CCNB2 promoted tumor growth of TNBC cells through an *in vivo* model. MDA-MB-231 cells were stably transfected with control or CCNB2 shRNA plasmids and subcutaneously injected into nude mice. After 14 days, tumors began formation; we measured the volume of tumors every 3 days. After 29 days, all tumors were isolated. The representative images of tumors and the tumor growth curves are shown in Figure 5(a). We found that the volume of CCNB2-depleted tumors was significantly decreased than that in control tumors. We further performed immunoblot assays to confirm the alteration of CCNB2 expression in tumor tissues from mice. We found that CCNB2 expression was decreased in CCNB2-depleted tumor tissues (Figure 5(b)). Collectively, we thought CCNB2 could contribute to tumor growth of TNBC cells in mice.

## 4. Discussion

Due to the lack of effective targeted therapy drugs, TNBC was an aggressive malignant tumor [15]. Also, the survival rates of TNBC patients are still the shortest among all breast cancer subtypes [16]. TNBC is prone to bone metastasis, and the liposome-mediated targeted therapy has a good effect [17, 18]. Currently, treatment for TNBC mainly includes surgical resection and chemoradiotherapy [19]. CAR-T immunotherapy also had the potential, but still at the initial stage [20]. Developing novel molecular targets and new promising drugs is urgently needed [21]. Interestingly, we identified that a cyclin protein, CCNB2, was abnormal highly expressed in human TNBC tissues. We further found the correlations between TNBC patients' prognosis, clinical pathological features, and CCNB2 expression in tumor tissues. According to these results, we thought CCNB2 could act as a promising therapeutic target for TNBC treatment.

Through the colony formation and MTT assays, we found that CCNB2 contributed to the proliferation of TNBC cells *in vitro*. Consistent with the *in vitro* data, we further noticed that CCNB2 promoted tumor growth of TNBC cells *in vivo*. Taken together, these studies demonstrated the involvement of CCNB2 in the progression of TNBC. Similarly, several studies indicated the critical role of CCNB2 in tumorigenesis and metastasis [12, 22]. CCNB2 was abnormally expressed in multiple types of tumors, such as lung cancer and gastric cancer, and correlated with the prognosis and clinical features of patients [23, 24]. CCNB2 was also overexpressed in human hepatocellular carcinoma (HCC) tissues and associated with the poor prognosis [10]. Additionally, CCNB2 was also a biomarker for the diagnosis of lung adenocarcinoma [24]. These studies confirmed the critical role of CCNB2 in cancer progression. Developing its specific inhibitors was a promising and usefulness manner for TNBC treatment.

As a cell cycle regulator, CCNB2 was involved in maintaining mitosis process [9]. It was known that the defects of CCNB2 led to the abnormal cell division and cell cycle arrest, and the expression of CCNB2 was also dependent on the cell cycle process [25]. We here found that CCNB2 could affect TNBC progression via regulating cell proliferation. Next, we should detect the effects of CCNB2 on cell cycle in TNBC cells. We here found the effects of CCNB2 on TNBC progression. In addition, we noticed that CCNB2 did not affect EMT process in TNBC cells, with the moderate effects on the expression of E-cadherin and N-cadherin (data not shown). We found that CCNB2 promoted tumor growth *in vivo*, and we further should assess the effects of CCNB2 on the migration, invasion, and apoptosis of TNBC cells.

Except for CCNB2, multiple cyclins were involved in the regulation of cancer progression and development [26]. Previous studies indicated that CCND1 participated in miR-502-5p-mediated suppression of cell proliferation and migration in bladder cancer [27]. Additionally, CCNA1 was associated with the poor prognosis of patients with oesophageal squamous cell carcinoma (OSCC) [28]. Another study demonstrated that CCNB1 regulated cell cycle progression in breast cancer [29]. These studies all demonstrated that cyclins could serve as promising molecular targets for cancer treatment. Developing inhibitors of the cyclins was a promising manner to combat cancers.

In this study, we found that CCNB2 was highly expressed in human TNBC tissues through IHC and bioinformatics analysis. However, the mechanism underlying the high expression of CCNB2 promoting TNBC progression is still unclear. Notably, CCNB2 has been reported for the abnormal expression in multiple types of cancers [23, 24]. CCNB2 expression was also increased in invasive breast carcinoma and associated with unfavorable clinical outcome [30].

The limitation of this study is that the tumor sample size is not large enough. Another key issue is that the mechanism by which CCNB2 regulates the progress of TNBC is still unknown. Although, as a cell cycle regulator, CCNB2 may further influence the development of TNBC through its influence on the cycle, we still need to confirm the signaling pathway through which CCNB2 influences the development of TNBC. In addition, we need to expand the number of tumor samples and conduct long-term follow-up on the survival of patients in order to further clarify the relationship between CCNB2 and TNBC. We need to design a series of experiments *in vitro* and *in vivo*, combined with transcriptome analysis, to clarify the molecular mechanism of CCNB2 regulation of TNBC.

Notably, previous studies showed that cell cycle regulators affected the progression of multiple types of tumors. Seven cell cycle-related genes have been identified with unfavorable prognosis of their TF-miRNA-mRNA regulatory network in breast cancer [31]. For TNBC, the correlation and mechanism between cell cycle regulators and TNBC progression need further in-depth study [32, 33].

In conclusion, we found the high expression of CCNB2 in human TNBC tissues, and the expression of CCNB2 was correlated with the prognosis and clinical features of TNBC patients. We further found that CCNB2 contributed to TNBC cell proliferation *in vitro* and in mice and therefore thought CCNB2 could serve as a promising molecular target for TNBC treatment.

## Figures and Tables

**Table 1:** Relationships of CCNB2 and clinicopathological characteristics in 114 patients with triple-negative breast cancer (TNBC).

Feature	All *n* = 114	CCNB2 expression		*χ* ^2^	*p*
		Low	High		
		*n* = 46	*n* = 68		
*Age (year)*				2.165	0.141
<50	64	22	42		
≥50	50	24	26		
*Tumor grade*				1.972	0.160
Low	66	23	43		
High	48	23	25		
*Tumor size*				5.258	0.022^∗^
<2	62	31	31		
≥2	52	15	37		
*pTNM stage*				5.329	0.021^∗^
I-II	75	36	39		
III-IV	39	10	29		
*Lymph node metastasis*				1.338	0.247
Yes	52	24	28		
No	62	22	40		

## Data Availability

The data used to support the findings of this study are available from the corresponding author upon request.
